# Exposure to Chinese famine and the risk of hyperuricemia in later life: a population-based cross-sectional study

**DOI:** 10.3389/fnut.2024.1266817

**Published:** 2024-01-17

**Authors:** Huali Xiong, Daiqiang Liu, Dayi Tang, Fengxun Ma

**Affiliations:** ^1^Department of Public Health, Health Commission of Rongchang District, Chongqing, China; ^2^Department of Hospital Information, The People's Hospital of Rongchang District, Chongqing, China; ^3^First Clinical College, Mudanjiang Medical College, Mudanjiang, Heilongjiang, China

**Keywords:** Chinese famine, fetal-exposed, childhood-exposed, adolescence-exposed, hyperuricemia

## Abstract

**Background:**

Limited studies have investigated the relationship between famine exposure and the risk of hyperuricemia in later life. Consequently, the primary purpose of the current study was to examine the potential association between exposure to Chinese famine and hyperuricemia, as well as any gender disparities in this relationship.

**Method:**

The data were obtained from the China PEACE (China Patient-Centered Evaluative Assessment of Cardiac Events) Million Persons Project in Rongchang. The study participants were enrolled into different cohorts based on their birthdates: the fetal-exposed cohort (born between 1959 and 1962), the childhood-exposed cohort (born between 1949 and 1958), the adolescence-exposed cohort (born between 1941 and 1948), and the non-exposed cohorts (born between 1963 and 1974). The potential association between famine exposure and hyperuricemia was assessed using binary logistic regression models.

**Results:**

A total of 6,916 individuals were enrolled in the current study with an average age of 60.11 ± 9.22 years, out of which 3,544 were women. After adjusting for confounding factors, fetal (*OR* = 0.530, 95% *CI*: 0.411–0.0.683), childhood (*OR* = 0.642, 95% *CI*: 0.494–0.833) exposure to the Chinese famine for men was negatively associated with hyperuricemia. Conversely, exposure to the Chinese famine during fetal (*OR* = 2.144, 95% *CI*: 1.622–2.834), childhood (*OR* = 1.485, 95% *CI*: 1.105–1.997), and adolescence (*OR* = 1.967, 95% *CI*: 1.465–2.641) for women was positively associated with hyperuricemia. Furthermore, the impact of famine on hyperuricemia that has been observed in exposed women might be intensified by the presence of dyslipidemia, abdominal obesity, and overweight/obesity.

**Conclusion:**

Women exposed to the Chinese famine during fetal, childhood, and adolescence were positively associated with hyperuricemia, while men exhibited a negative association during fetal and childhood. Additionally, the effect of famine on hyperuricemia in exposed women appears to be intensified by the presence of dyslipidemia, abdominal obesity, and overweight/obesity.

## Introduction

Hyperuricemia (HUA) is a cardiometabolic risk factor/metabolic disease resulting from a disorder in purine metabolism (1). With the vigorous economic development and large-scale urbanization in mainland China, the prevalence of hyperuricemia has reached 17.4% (2). In comparison, the prevalence of hyperuricemia in the United States, Japan, and Korea was 14.60%, 13.4%, and 11.4% (3–5), respectively. Additionally, previous studies have demonstrated that hyperuricemia is linked with hypertension, type 2 diabetes mellitus, kidney disease, and coronary artery disease (6, 7).

Hyperuricemia has emerged as the second largest metabolic disease, ranking next to diabetes mellitus in mainland China (8). Factors including age, gender, ethnicity, genetics, smoking, alcohol consumption, obesity, physical activity, and dietary patterns have been identified as contributors to the development of hyperuricemia (2, 8, 9). Previous studies have found an elevated risk between early-life exposure to Chinese famine and metabolic syndrome (10, 11). Building upon these insights, we hypothesize that famine exposure is associated with hyperuricemia.

It was possible to study the effects of famine exposure and health outcomes in later life during the Chinese famine, which occurred between 1959 and 1962. Previous studies have demonstrated that early-life exposure to Chinese famine makes people more likely to develop hypertension (12), diabetes mellitus (13), dyslipidemia (14), and obesity (15) in adulthood. However, there remains a dearth of studies on the association (16, 17) between famine exposure and hyperuricemia, which has not been fully elucidated. Additionally, there is a notable absence of research on the relationship between exposure to the Chinese famine during adolescence and hyperuricemia in the general population. Moreover, the gender differences in the association between famine exposure and hyperuricemia deserve to be studied. A comprehensive understanding of this association holds potential benefits for the prevention of hyperuricemia. To address these gaps in knowledge, the present study utilizes the baseline data from the China Patient-Centered Evaluative Assessment of Cardiac Events Million Persons Project in Rongchang to explore the relationship between exposure to Chinese famine and hyperuricemia, with a specific focus on gender differences.

## Methods

### Study population

**Figure 1:**
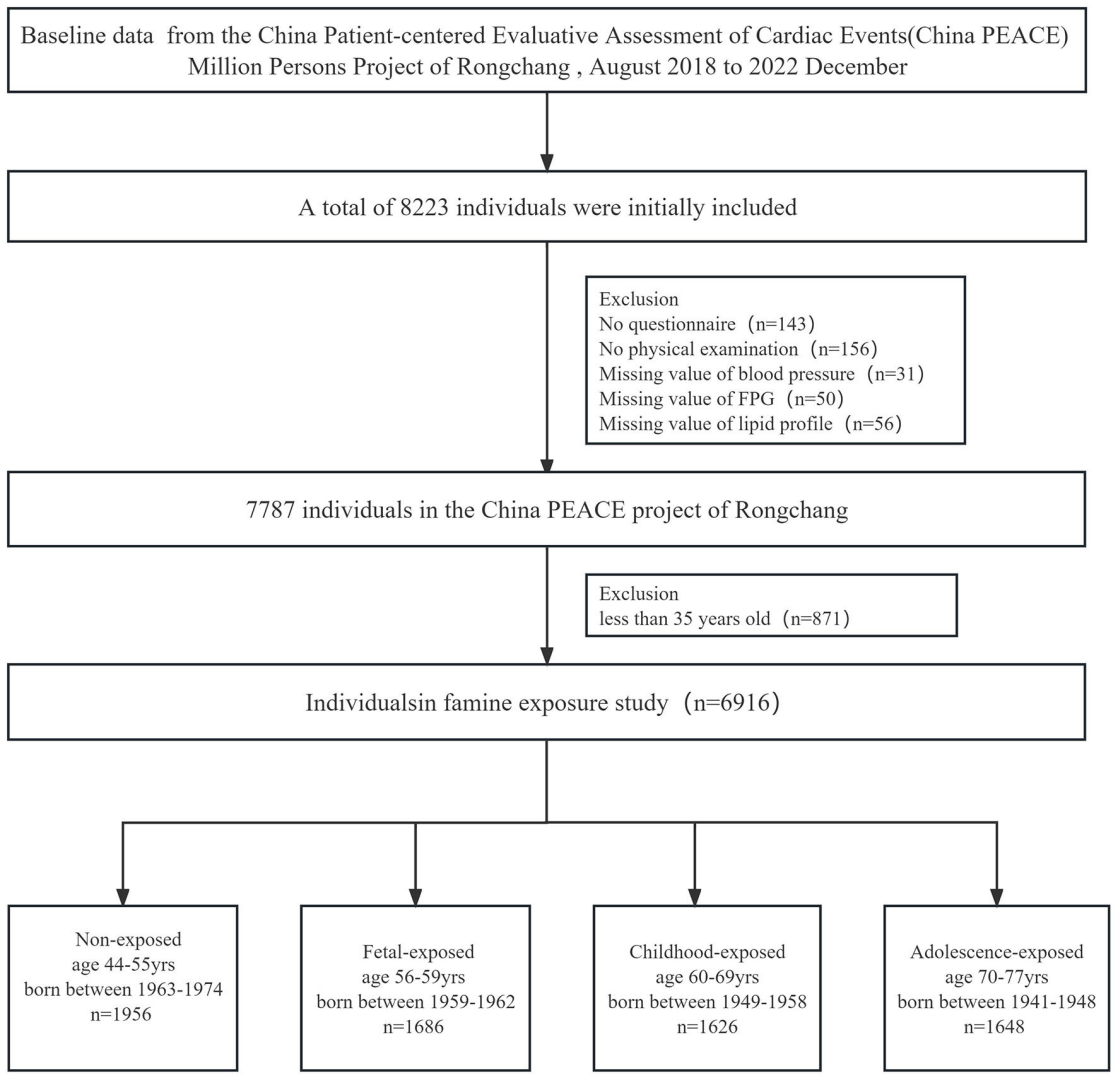
Flowchart on the sample selecting method at each step.

The China PEACE (China Patient-Centered Evaluative Assessment of Cardiac Events) Million Persons Project is a nationwide cardiovascular disease screening and comprehensive intervention project. The design and details of the China PEACE Project have been described elsewhere (18). This study was conducted in Rongchang between August 2018 and December 2022 using a three-stage stratified random sampling method. Details can be seen in a previous study (14). The initial recruitment included 8,223 individuals from four streets, namely, Chang yuan, Chang zhou, Guang shun, and An fu. Specific inclusion and exclusion criteria were implemented to ensure the study's reliability and validity. Briefly, in Rongchang, participant inclusion criteria were (1) age between 35 and 75 years, (2) registered address in Rongchang, (3) living in Rongchang for at least 6 months, (4) Han ethnicity, (5) the absence of cognitive dysfunction, disturbance of consciousness, or impaired communication abilities. Participants were excluded from the study if they met any of the following criteria: (1) age under 35 or over 75 years, (2) those who were unable to participate in all surveys, (3) the presence of missing information on questionnaires, physical examinations, and blood biochemical tests. In total, 1,307 participants were excluded from the current study based on the specified exclusion criteria (Figure 1). This study had been approved by both the Ethics Committee of the Rongchang Center for Disease Control and Prevention (no. RCJK20180023) and the Central Ethics Committee at the China National Center for Cardiovascular Disease (no. 2014–574). All participants were fully informed about the research's purpose and precautions, and they signed informed consent forms before the investigation.

### Famine definition

The famine cohorts in the current study were established based on birthdate: fetal-exposed cohorts (born between 1959 and 1962), childhood-exposed cohorts (born between 1949 and 1958), adolescence-exposed cohorts (born between 1941 and 1948), and non-exposed cohorts (born between 1963 and 1974). The same classification based on exposure to Chinese famine has been utilized in previous studies (14, 19), ensuring consistency and comparability across research. In this study, a total of 6,916 individuals were included, representing the various famine cohorts mentioned above.

### Study outcomes

The primary objective of this study was to examine the gender difference in the relationship between famine exposure and the risk of hyperuricemia in later life. Basic demographic characteristics of participants were collected and recorded, such as age, gender, and ethnicity. Behavior and lifestyle information, including smoking habits, alcohol consumption, and physical activity levels, were gathered through structured questionnaires. Trained general practitioners with at least 10 years of experience conducted the comprehensive physical examinations on the study participants. Blood biochemical tests were performed by skilled laboratory physicians to assess various biomarkers, including uric acid levels, glucose, and lipids.

### Assessment of covariates

The current study considered several covariates to assess their potential influence on the association between famine exposure and the risk of hyperuricemia in later life, including gender, age, marital status (married/cohabitated or others), job condition (farmers or others), educational status (primary school or below, junior middle school or high school, or above), average yearly income (< 20,000 yuan, 20,001–59,999 yuan, or ≥60,000 yuan), smoking status (former/current or never), and drinking status (former/current or never). Smoking and drinking status were collected by a positive answer to this question: “Did you ever smoke or drink alcohol a month or so before.” The physical activity level was classified into three groups (light, moderate, and vigorous) according to the Chinese guidelines for data processing and analysis concerning the International Physical Activity Questionnaire (20). Standard physical examinations were conducted to measure participants' height, weight, and waist circumference (WC). Participants in a resting position underwent three measurements for systolic/diastolic blood pressure (SBP and DBP), at 5-min intervals using an electronic sphygmomanometer (Omron HBP-1300, China). Fingertip blood samples were collected to detect fast blood glucose (FBG) and uric acid (UA) levels using a monitoring system (BeneCheck PD-G001–2, China). Lipid profiles were measured using a rapid blood lipid detector (Cardiocheck PA Analyzer, United States), including total cholesterol (TC), triglycerides (TG), high-density lipoprotein cholesterol (HDL-C), and low-density lipoprotein cholesterol (LDL-C). Hypertension (21) (HTN) was defined as having an SBP ≥140 mmHg and/or DBP ≥90 mmHg, or self-reported physician-diagnosed HTN, or taken measures (medications and lifestyle changes) for HTN. Diabetes mellitus (22) (DM) was defined as having a FPG≥7.0 mmol/L, self-reported physician-diagnosed DM, or taking measures (medication and lifestyle changes) for DM. Dyslipidemia (23) was defined as the presence of any of the following criteria: TC≥6.22 mmol/L or/and TG>2.30 mmol/L or/and HDL-C≥4.10 mmol/L or/and LDL-C < 1.00 mmol/L. Body mass index (15) (BMI) was calculated using height and weight; BMI is determined by dividing weight by the square of height and categorized into three groups (“ < 24,” “24–27.9,” and “≥ 28”; and BMI ≥ 28 kg/m^2^ was defined as obesity). Abdominal obesity (15) was defined as having a WC ≥ 90 cm for men and a WC≥85 cm for women, following the previous study on abdominal obesity and Chinese famine. According to the Chinese expert consensus on the treatment of hyperuricemia and gout, serum uric acid concentrations >420 μmol/L for men and >360 μmol/L for women were defined as hyperuricemia (24) (HUA).

### Statistical analysis

Means ± standard deviation (S.D.) is used for continuous variables that conform to a normal distribution, while median and quartile [M (P25, P75)] are used for continuous variables with a non-normal distribution. We compared the general characteristics of individuals stratified by gender according to HUA using the chi-squared test and the Student's *t*-test. Additionally, we compared the general characteristics of the study participants based on their exposure status to the Chinese famine using the chi-squared test and one-way ANOVA. Binary logistic regression models were employed to calculate the OR (odds ratio) and 95% CI (confidence interval) of the association between famine exposure and HUA. In the analysis, Model 1 was the unadjusted model. Model 2 was adjusted for various confounding factors, including age, marital status, job conditions, educational status, average yearly income, smoking and drinking status, physical activity, HTN, DM, dyslipidemia, abdominal obesity, and BMI. Model 3 served as a sensitivity analysis model, which excluded individuals with HTN, DM, dyslipidemia, abdominal obesity, and BMI ≥ 28 kg/m^2^. Model 3 was adjusted for confounding factors including age, marital status, job conditions, educational status, average yearly income, smoking and drinking status, and physical activity. Furthermore, we analyzed the multiplicative interaction of famine exposure and various health conditions, such as HTN, DM, dyslipidemia, abdominal obesity, and BMI using the likelihood ratio test. To mitigate the impact of age differences on the correlation between famine exposure and the risk of hyperuricemia, we employed four different methods as an age-balanced control strategy for our analysis. Initially, we conducted univariate and multivariate logistic regression analyses to examine the associations between age and hyperuricemia. Subsequently, we employed simple linear regression to investigate the relationship between different birth years of famine exposure and the prevalence of hyperuricemia. Finally, we utilized age-matched control groups, comprising the unexposed (1963–1974), younger unexposed (1975–1988), and unexposed combing (1963–1974) and (1975–1988) as references to seek the association between fetal, childhood, and adolescent exposure to the Chinese famine and the risk of hyperuricemia. Also, we combined the unexposed (1963–1974) and adolescence-exposed (1941–1948) groups as a reference category, which was instrumental in scrutinizing the association between fetal and childhood exposed groups and the subsequent risk of hyperuricemia in later life. SPSS version 26.0 was applied for statistical analyses and a *P* < 0.05 was considered statistical significance.

## Results

### Characteristics of study participants stratified by gender

Characteristics of individuals stratified by gender according to HUA are presented in Table 1. In this study, 6,919 individuals were included, with an average age of 60.11 ± 9.22 years. Among them, 3,372 men and 3,544 women were exposed to the Chinese famine during 1959–1962. The prevalence of hyperuricemia in men and women was 19.99% and 20.82%, respectively. However, the difference in prevalence was not statistically significant (χ^2^ = 0.743, *P* = 0.389). Regarding specific characteristics, men with hyperuricemia are more likely to be smokers and alcohol users. The mean age of women with hyperuricemia was greater than that of men with hyperuricemia. Furthermore, the prevalence of hyperuricemia in men with hypertension, diabetes mellitus, dyslipidemia, obesity, and abdominal obesity was higher than those in women.

**Table 1:** General characteristics of individuals stratified by gender according to HUA.

**Characteristics**	**Male (*****n** =* **3,372)**			**Female (** ***n** =* **3,544)**			**t/χ^2^**	***P***
	**Total**	**HUA**	**%**	**Total**	**HUA**	**%**		
Number	3,372	674	19.99	3,544	738	20.82	χ^2^ = 0.743	0.389
Age in survey, years	58.90 ± 9.12			61.27 ± 9.17			t = −10.806	< 0.001
Education level							χ^2^ = 5.507	0.019
Primary school or below	808	150	18.56	1,082	258	23.84		
Junior middle school	1,078	224	20.78	1,288	278	21.58		
High school or above	1,486	300	20.19	1,174	202	17.21		
Marital status							χ^2^ = 0.661	0.260
Married/cohabitated	3,154	624	19.78	2,780	578	20.79		
Others	218	50	22.94	764	160	20.94		
Job conditions							χ^2^ = 0.105	0.746
Farmers	1,200	212	17.67	1,556	356	22.88		
Others	2,172	462	21.27	1,988	382	19.22		
Average yearly income, yuan							χ^2^ = 41.354	< 0.001
< 20,000	1,118	196	17.53	1,432	344	24.02		
20,001–59,999	1,242	232	18.68	1,282	189	14.74		
≥60,000	1,012	246	24.31	830	205	24.70		
Smoking							χ^2^ = 0.214	0.643
Never	1,972	396	20.08	3,544	738	20.82		
Former/current	1,400	278	19.86	-	-	-		
Drinking							χ^2^ = 2.879	0.090
Never	1,294	210	16.23	2,412	575	23.84		
Former/current	2,078	464	22.33	1,132	163	14.40		
Physical activity							χ^2^ = 21.487	< 0.001
Light	552	138	25.00	892	197	22.09		
Moderate	648	154	23.77	920	202	21.96		
Vigorous	2,158	376	17.42	1,724	339	19.66		
Hypertension							χ^2^ = 74.314	< 0.001
No	1,564	234	14.96	1,580	264	16.71		
Yes	1,808	440	24.34	1,964	474	24.13		
Diabetes							χ^2^ = 4.199	0.040
No	2,880	572	19.86	3,000	653	21.77		
Yes	492	102	20.73	544	85	15.63		
Dyslipidemia							χ^2^ = 98.322	< 0.001
No	2,592	473	18.25	2,434	405	16.64		
Yes	780	201	25.77	1,110	333	30.00		
BMI group, kg/m^2^							χ^2^ = 49.335	< 0.001
< 24	1,272	220	17.30	1,324	196	14.80		
≥24	2,100	454	21.62	2,220	542	24.41		
Abdominal obesity							χ^2^ = 97.146	< 0.001
No	2,390	400	16.74	2,150	370	17.21		
Yes	982	274	27.90	1,394	368	26.40		

General characteristics of individuals classified by gender were evaluated using t-test or χ^2^-test. %: the prevalence of HUA in different characteristics.

### Characteristics of study participants stratified by their exposure to the Chinese famine

Characteristics of individuals based on their Chinese famine exposure status are presented in Table 2. In total, 1,686, 1,626, and 1,648 individuals were exposed to Chinese famine during fetal, childhood, and adolescence. The distribution of age, gender, marital status, education level, job conditions, average yearly income, smoking status, drinking status, physical activity level, HTN, DM, dyslipidemia, abdominal obesity, and BMI demonstrated statistically significant differences among the four cohorts (all *P* < 0.05).

**Table 2:** General characteristics of the study participants according to the Chinese famine exposure.

**Variables**	**N**	**Non-exposed**	**Fetal-exposed**	**Childhood-exposed**	**Adolescence-exposed**	**F/χ^2^**	***P***
		**(1963–1974)**	**(1959–1962)**	**(1949–1958)**	**(1941–1948)**		
		**(*****n** =* **1,956)**	**(*****n** =* **1,686)**	**(*****n** =* **1,626)**	**(*****n** =* **1,648)**		
Age	6,916	48.73 ± 3.39	57.22 ± 1.16	63.92 ± 2.65	72.65 ± 2.19	*F* = 2,847.204	< 0.001
Gender							
Male	3,372	1,176 (60.12)	800 (47.45)	730 (44.90)	666 (40.41)	χ^2^ = 157.918	< 0.001
Female	3,544	780 (39.88)	886 (52.55)	896 (55.10)	982 (59.59)		
Marital status							
Married/cohabitated	5,934	1,822 (93.15)	1,474 (87.43)	1,424 (87.58)	1,214 (73.67)	χ^2^ = 293.788	< 0.001
Others	982	134 (6.85)	212 (12.57)	202 (12.42)	434 (26.33)		
Education level							
Primary school or below	1,890	346 (17.69)	616 (36.54)	472 (29.03)	456 (27.67)	χ^2^ = 545.257	< 0.001
Junior middle school	2,366	602 (30.78)	332 (19.69)	636 (39.11)	796 (48.30)		
High school or above	2,660	1,008 (51.53)	738 (43.77)	518 (31.86)	396 (24.03)		
Job conditions							
Farmers	2,756	688 (35.17)	596 (35.35)	706 (43.42)	766 (46.48)	χ^2^ = 70.958	< 0.001
Others	4,160	1,268 (64.83)	1,090 (64.65)	920 (56.58)	882 (53.52)		
Average yearly income							
< 20,000 yuan	2,550	622 (31.80)	634 (37.60)	650 (39.98)	644 (39.08)	χ^2^ = 91.159	< 0.001
20,001–59,999 yuan	2,524	766 (39.16)	522 (30.96)	568 (34.93)	668 (40.53)		
≥60,000 yuan	1,842	568 (29.04)	530 (31.44)	408 (25.09)	336 (20.39)		
Smoking							
Never	5,514	1,472 (75.26)	1,266 (75.09)	1,350 (83.03)	1,426 (86.53)	χ^2^ = 104.762	< 0.001
Former/current	1,402	484 (24.74)	420 (24.91)	276 (16.97)	222 (13.47)		
Drinking							
Never	3,706	872 (44.58)	858 (50.89)	888 (54.61)	1,088 (66.02)	χ^2^ = 171.826	< 0.001
Former/current	3,210	1,084 (55.42)	828 (49.11)	738 (45.39)	560 (33.98)		
Physical activity							
Light	1,466	204 (10.44)	336 (19.93)	458 (28.27)	446 (27.29)	χ^2^ = 754.996	< 0.001
Moderate	1,568	222 (11.36)	308 (18.27)	480 (29.63)	558 (34.15)		
Vigorous	3,882	1,528 (78.20)	1,042 (61.80)	682 (42.10)	630 (38.56)		
Hypertension							
No	3,144	1,218 (62.27)	812 (48.16)	624 (38.38)	490 (29.73)	χ^2^ = 425.194	< 0.001
Yes	3,772	738 (37.73)	874 (51.84)	1,002 (61.62)	1,158 (70.27)		
Diabetes							
No	5,880	1,786 (91.31)	1,382 (81.97)	1,350 (83.03)	1,362 (82.65)	χ^2^ = 85.434	< 0.001
Yes	1,036	170 (8.69)	304 (18.03)	276 (16.97)	286 (17.35)		
Dyslipidemia							
No	5,026	1,548 (79.14)	1,162 (68.92)	1,138 (69.99)	1,178 (71.48)	χ^2^ = 60.243	< 0.001
Yes	1,890	408 (20.86)	524 (31.08)	488 (30.01)	470 (28.52)		
Abdominal obesity							
No	4,540	1,470 (75.15)	1,156 (68.56)	1,014 (62.36)	900 (54.61)	χ^2^ = 181.515	< 0.001
Yes	2,376	486 (24.85)	530 (31.44)	612 (37.64)	748 (45.39)		
BMI (kg/m^2^)							
< 24	2,596	738 (37.73)	648 (38.43)	526 (32.35)	684 (41.50)	χ^2^ = 30.339	< 0.001
≥24	4,320	1,956 (28.28)	1,686 (24.38)	1,626 (23.51)	1,648 (23.83)		
UA level, μmol/L	6,916	324 (267,380)	326 (261,384)	314 (267,371)	327 (270,377)	H = 12.722	0.005
Hyperuricemia							
No	5,504	1,612 (29.29)	1,356 (24.64)	1,307 (23.75)	1,229 (22.33)	36.454	< 0.001
Yes	1,412	344 (24.36)	330 (23.37)	319 (22.59)	419 (29.67)		

Distribution of the four exposed cohorts was evaluated using t-test or χ^2^-test or one-way ANOVA.

### The association between exposure to the Chinese famine and hyperuricemia

The association between famine exposure and hyperuricemia is presented in Table 3. After adjusting for confounding factors (Model 2), men exposed to the Chinese famine during fetal (*OR* = 0.530, 95% CI: 0.411–0.0.683), childhood(*OR* = 0.642, 95% CI: 0.494–0.833) were negatively associated with hyperuricemia, while women exposed to the Chinese famine during fetal (*OR* = 2.144, 95% *CI*: 1.622–2.834), childhood (*OR* = 1.485, 95% *CI*: 1.105–1.997), and adolescence (*OR* = 1.967, 95% *CI*: 1.465–2.641) were positively associated with hyperuricemia.

**Table 3:** The association between exposure to the Chinese famine and hyperuricemia.

**Variables**	**Non-exposed**	**Fetal-exposed**	**Childhood-exposed**	**Adolescent-exposed**
Male				
Model	Ref.	0.654 (0.515–0.830)^**^	0.803 (0.634–1.016)	1.310 (1.049–1.636)^*^
Mode 2	Ref.	0.530 (0.411–0.683)^**^	0.642 (0.494–0.833)^**^	1.178 (0.915–1.516)
Mode 3	Ref.	0.866 (0.525–1.429)	0.283 (0.137–0.587)^**^	0.429 (0.215–0.855)^**^
Female				
Model	Ref.	2.267 (1.793–2.956)^**^	1.951 (1.491–2.552)^**^	2.426 (1.871–3.145)^**^
Mode 2	Ref.	2.144 (1.622–2.834)^**^	1.485 (1.105–1.997)^**^	1.967 (1.465–2.641)^**^
Mode 3	Ref.	1.258 (0.614–2.575)	3.067 (1.527–6.160)^**^	7.836 (3.858–15.918)^**^

Evaluating the gender risk of three exposed cohorts with non-exposed as a reference by the binary logistic regression model. Model 1: unadjusted. Model 2: adjusted for age, marital status, job conditions, educational status, average yearly income, smoking and drinking status, physical activity, hypertension, diabetes, dyslipidemia, abdominal obesity, and BMI. Model 3: exclusion of the individuals with hypertension, diabetes, dyslipidemia, abdominal obesity and BMI≥28, adjusted for age, marital status, job conditions, educational status, average yearly income, smoking and drinking status, and physical activity. ^*^P < 0.05; ^**^P < 0.01; CI, confidence interval; Ref., defined as reference.

### Sensitivity analysis

In the sensitivity analysis, 5,348 participants with HTN, DM, dyslipidemia, obesity, and abdominal obesity were excluded from the current study. After adjusting for confounding factors (Model 3), men exposed to the Chinese famine during childhood (*OR* = 0.283, 95% *CI*: 0.137–0.587), adolescence (*OR* = 0.429, 95% *CI*: 0.215–0.855) were negatively associated with hyperuricemia, women exposed to the Chinese famine during childhood (OR = 3.067, 95% CI: 1.527–6.160) and adolescence (OR = 7.836, 95% CI: 3.858–15.918) were positively associated with hyperuricemia.

### Stratified analyses

Multivariable-adjusted *OR*s (95% CI) for the association of HTN, DM, dyslipidemia, abdominal obesity, BMI, and famine exposure with hyperuricemia are presented in Table 4. The interaction existed between famine exposure and dyslipidemia, abdominal obesity, and BMI in women. Compared with the non-exposed female participants without dyslipidemia, the presence of dyslipidemia in conjunction with fetal-exposed (*OR* = 6.604, 95% *CI*: 4.565–9.553), childhood-exposed (*OR* = 2.501, 95% *CI*: 1.672–3.74), and adolescence-exposed (*OR* = 3.696, 95% *CI*: 2.482–5.505) participants had an increased risk of hyperuricemia. A similar association was observed between abdominal obesity, BMI, and famine exposure with hyperuricemia in female participants.

**Table 4:** Multivariable-adjusted *OR*s (95%*CI*) for the association of HTN, DM, dyslipidemia, abdominal obesity, BMI, and famine exposure with hyperuricemia.

**Variables**	**Male**				
	**Non-exposed**	**Fetal-exposed**	**Childhood-exposed**	**Adolescent-exposed**	**P**_interaction_
Hypertension					0.435
No	1.000	0.659 (0.446–0.973)	0.493 (0.326–0.743)	0.967 (0.626–1.494)	
Yes	1.484 (1.110–1.983)	0.723 (0.523–0.999)	1.111 (0.803–1.538)	1.918 (1.426–2.579)	
Diabetes					
No	1.000	0.579 (0.440–0.762)	0.673 (0.509–0.890)	1.073 (0.817–1.409)	0.236
Yes	0.829 (0.520–1.321)	0.331 (0.198–0.551)	0.447 (0.259–0.773)	1.674 (1.041–2.693)	
Dyslipidemia					
No	1.000	0.608 (0.456–0.811)	0.612 (0.453–0.827)	0.923 (0.686–1.243)	0.534
Yes	1.179 (0.844–1.649)	0.437 (0.281–0.679)	0.825 (0.543–1.255)	2.448 (1.671–3.585)	
Abdominal obesity					
No	1.000	0.798 (0.592–1.076)	0.637 (0.461–0.881)	1.006 (0.735–1.376)	0.223
Yes	1.787 (1.304–2.448)	0.420 (0.269–0.654)	1.166 (0.794–1.711)	2.739 (1.912–3.922)	
BMI>24					
No	1.000	1.059 (0.696–1.613)	0.533 (0.355–0.850)	1.103 (0.741–1.640)	0.452
Yes	1.133 (0.814–1.577)	0.421 (0.284–0.624)	0.754 (0.516–1.103)	1.302 (0.884–1.918)	
**Variables**	**Female**				
	**Non-exposed**	**Fetal-exposed**	**Childhood-exposed**	**Adolescent-exposed**	**P**_interaction_
Hypertension					0.332
No	1.000	2.730 (1.876–3.971)	2.015 (1.322–3.071)	1.545 (0.983–2.430)	
Yes	1.417 (0.896–2.240)	2.472 (1.698–3.598)	1.790 (1.241–2.582)	3.063 (2.132–4.400)	
Diabetes					
No	1.000	2.202 (1.631–2.973)	1.728 (1.267–2.355)	2.412 (1.764–3.298)	0.091
Yes	1.473 (0.716–3.031)	1.511 (0.948–2.407)	0.402 (0.215–0.750)	0.522 (0.303–0.898)	
Dyslipidemia					
No	1.000	1.700 (1.182–2.445)	1.816 (1.266–2.605)	2.184 (1.531–3.115)	0.012
Yes	2.469 (1.527–3.993)	6.604 (4.565–9.553)	2.501 (1.672–3.74)	3.696 (2.482–5.505)	
Abdominal obesity					0.035
No	1.000	3.277 (2.335–4.599)	1.664 (1.137–2.435)	1.821 (1.232–2.691)	
Yes	2.150 (1.339–3.453)	1.694 (1.081–2.654)	2.708 (1.868–3.926)	3.970 (2.794–5.641)	
BMI>24					0.023
No	1.000	4.838 (2.762–8.476)	2.136 (1.119–4.077)	2.687 (1.482–4.871)	
Yes	3.386 (1.918–5.977)	4.769 (2.740–8.301)	4.137 (2.373–7.213)	5.295 (3.036–9.235)	

OR, odds ratio; CI, confidence interval.

### Age balance control

The influencing factors associated with hyperuricemia are detailed in Supplementary Tables S1 and S2. Age demonstrated a positive association with hyperuricemia in univariate logistic regression analyses (*OR* = 1.018, 95% *CI*: 1.012–1.025), while no association was found in multivariate logistic regression (*OR* = 0.999, 95% *CI*: 0.975–1.024). The simple linear regression model revealed no relationship between different birth years of famine exposure and the prevalence of hyperuricemia (Supplementary Figure 1). We observed that odds ratios (*OR*s) can vary depending on the combinations of birth years used as controls after adjusting for confounding factors (Model 2). The association between fetal and childhood exposure to the Chinese famine and the risk of hyperuricemia compared with age-matched control groups in later life is presented in Supplementary Table S3. Compared with the age-matched control group, men exposed to the Chinese famine during the fetal (*OR* = 0.590, 95% *CI*: 0.473–0.737) and childhood (*OR* = 0.725, 95% *CI*: 0.582–0.902) periods exhibited a negative association with hyperuricemia. In contrast, women exposed to the Chinese famine during fetal (*OR* = 1.073, 95% *CI*: 1.073–1.585) and childhood (*OR* = 1.290, 95% *CI*: 1.057–1.576) showed a positive association with hyperuricemia (Supplementary Table S3).

## Discussion

In the present study, using data from the China PEACE Million Persons Project in Rongchang, women exposed to the Chinese famine during fetal, childhood, and adolescence were positively associated with hyperuricemia. In contrast, the association among male participants was negative during fetal and childhood. Furthermore, the study findings indicate that the effect of famine on hyperuricemia might be intensified by the presence of dyslipidemia, abdominal obesity, and overweight/obesity in exposed women. These findings contribute to a deeper understanding of the complex relationships between famine exposure and the risk of hyperuricemia.

The relationship between early-life exposure to the Chinese famine and hyperuricemia has been reported, but there remains a lack of sufficient evidence concerning the direct link between famine exposure and hyperuricemia. Previous studies have provided some indications that fetal exposure to famine is associated with an increased risk of hyperuricemia in adulthood (16, 25). Wang et al. (17) found that individuals exposed to the Chinese famine during fetal and childhood with high economic status exhibited a positive association between famine exposure and hyperuricemia. This finding suggests that socioeconomic factors may interact with famine exposure to influence the risk of hyperuricemia in early-life-exposed populations. However, it is important to note that the previous studies mentioned above were limited in their scope, as they focused solely on early-life exposure to the Chinese famine and did not explore potential gender differences in the relationship between famine exposure and hyperuricemia. Realizing the gender-specific effects is crucial, as men and women may respond differently to famine exposure due to physiological and hormonal differences. Zhang et al. (26) found that exposure to Chinese famine during fetal, childhood, and adolescence was associated with a higher chance of having hyperuricemia in the hypertensive population. Shao et al. (27) provided interesting findings regarding the association between famine exposure and hyperuricemia. According to their results, women exposed to Chinese famine during childhood and adolescence was associated with an increased risk of hyperuricemia, while no significant association was observed during the fetal period. On the other hand, there was no significant association during fetal, childhood, or adolescence in men. It is essential to consider certain limitations of the Shao's study. First, the study was conducted in Qingdao during 2006–2009, which was too outdated. Additionally, being a coastal city, long-term intake of purine foods could potentially impact the association between famine exposure and hyperuricemia, as purine-rich foods can influence uric acid levels. The observed differences in different studies could be attributed to various factors, including differences in study design, the definition of non-exposed and exposed cohorts, the sampling method for participant selection, and the approach used to adjust for different confounding factors.

Our findings on the aggravating effects of dyslipidemia, abdominal obesity, and overweight/obesity on hyperuricemia in exposed women provide valuable insights into the mechanisms underlying this relationship. While the mechanism of hyperuricemia caused by dyslipidemia is not fully understood, research by Peng et al. (28) demonstrated that higher TG, TC, and LDL-c were significantly associated with elevated serum uric acid levels. High BMI and abdominal obesity are considered to be associated with insulin resistance, which can promote the reabsorption of uric acid by increasing the sodium hydrogen exchanger in the renal tubules (29–31). In addition, impaired uric acid clearance and the influence of hyperinsulinemia secondary to insulin resistance (29) may be the primary factors contributing to hyperuricemia in obese individuals. Importantly, previous studies demonstrated that women exposed to Chinese famine increased the risk of dyslipidemia (14) and overweight/obesity (32) in the Chongqing population. This observation aligns with the thrifty phenotype hypothesis (33), the occurrence of pathological changes after early malnutrition may be decided by the superposition of risk factors in later life.

Why is there a negative relationship between famine exposure and hyperuricemia in men? This might be linked with son preference (34) and mortality advantage (34, 35). In ancient Chinese traditional culture, sons were always favored and valued more than daughters, which may have led to unequal food distribution within families. Men were well nourished when they suffered from famine, while women might be sold to wealthy families in exchange for more survival opportunities for men. Even if women escaped from famine, they would experience long periods of food shortages (36). Another possible reason for the gender difference may be a mortality advantage. During famine periods, the mortality in men was higher than that in women, men who survived famine were more likely to be healthier and better equipped to endure the challenges of malnutrition and food scarcity (34), which is a common viewpoint that aligns with Darwin's survival theory (37).

The mechanism linking famine exposure to hyperuricemia remains presently unclear. The Fetal Origins of Adult Disease (FOAD) (38) hypothesis posits that the response and adaptation of fetuses to a malnourished *in-utero* environment can induce permanent procedural changes in the body's organ and tissue structure, physiology, and metabolism. These alterations are believed to exert lifelong effects on individuals. Further research (39) has indicated that malnutrition during all stages of development may contribute to chronic diseases later in life. Malnutrition could potentially influence the development of less vital organs (e.g., pancreas, liver, kidneys) to prioritize the protection of more critical organs, such as the brain (17). Early-life exposure to famine directly resulted in intrauterine growth restriction. The low birth weight group exhibited a 20% decrease in the number of nephrons compared to the normal group, along with a 10% increase in serum creatinine clearance (40). Birth weight showed a significant positive correlation with the number and weight of glomeruli while demonstrating a significant negative correlation with the volume of glomeruli (40). Early malnutrition is linked to the developmental disruption of the Notch signaling pathway—an essential route for nephrin formation. The compromised signaling pathway results in a reduction in the number of nephrons (41). Animal studies have demonstrated an association between a reduction in the number of nephrons and the occurrence of renal dysfunction (42, 43). Participants who underwent malnutrition may exhibit impaired kidney function and reduced uric acid excretion in later life (17). Furthermore, malnutrition activates the hypothalamic-pituitary-adrenal axis (44), leading to an excessive production of uric acid (27). Women exposed to prolonged food shortages during critical periods of growth and development may experience a reduction in the secretion of gonadotropin-releasing hormone, significantly impacting their metabolic abilities. Specifically, the decline in female estrogen secretion contributes to a decrease in uric acid excretion, elevating the risk of hyperuricemia in women (45).

It is imperative to thoroughly consider age as a confounding factor in the investigation of the relationship between famine exposure and health outcomes in adulthood. Consequently, we employed various methods to analyze the impact of age, ensuring the reliability of our findings. Initially, we examined the association between age and hyperuricemia using both univariate and multivariate analyses. Our findings revealed that age positively influenced hyperuricemia without adjusting for confounding factors in univariate analyses. Subsequently, we explored the relationship between birth year and the prevalence of hyperuricemia using a simple linear regression model. The R-squared value was 0.131, indicating that only 13.1% of the total association could be attributed to age, with a *p* > 0.05. Additionally, we employed different control groups to investigate changes in Odds Ratios (*OR*s) across famine-exposed groups. Notably, the *OR*s consistently increased, and our analysis indicated that the selection of the non-exposed group (1963–1974) as a control was optimal. Finally, we assessed the association between fetal and childhood exposure and hyperuricemia, utilizing the combined non-exposed group (1963–1974) and the adolescence-exposed group (1941–1948) as a control. Our findings indicated a similar association between fetal and childhood exposure to the Chinese famine and the risk of hyperuricemia in later life. Consequently, we have confidence in the robustness of our study results.

The Chinese famine had widespread effects on the entire mainland of China. However, its severity varied considerably across provinces due to differences in population density, local food shortages, and weather conditions. Studies on famine in China typically employ excess mortality rates (EMR) to gauge the severity (46). A severe famine area is defined by an EMR exceeding 50%, whereas less severe famine areas have an EMR below this threshold (46). Stratified analyses are employed to investigate whether a consistent association exists between famine exposure and health outcomes in areas with severe and less severe famines. According to the Liu et al. (47), the EMR in Chongqing ranged from 14.90% to 25.50%, categorizing Chongqing as a less severe famine area. However, recent studies have increasingly utilized the cohort size shrinkage index (CSSI) compared to the EMR. Both Liu et al. (46) and Chen et al. (48) employed CSSI to enhance the robustness of their studies. In Liu et al. (49), the CSSI in Chongqing exceeded 60%, designating it as a severe famine area. Liu et al. (46) study utilized data from the China Nutrition and Health Survey, a national cross-sectional survey on nutrition and diseases. On the other hand, Chen et al. (48) study focused on the entire Sichuan province, allowing for the use of different CSSIs at the province and prefecture levels for robustness analysis. It is noteworthy that Chongqing became a separate municipality from Sichuan Province after 1997, similar to Beijing, Tianjin, and Shanghai. Rongchang, a district in Chongqing, lacked individual-level data on famine intensity, leading to its exclusion from our analysis. Nonetheless, we recommend that famine studies in China comprehensively consider the impact of age and famine intensity.

Our study indeed has several notable strengths. First, to our knowledge, this is the first study to examine the association between exposure to Chinese famine and hyperuricemia and elucidate the gender difference in this association using general populations with a large sample size. Second, equally trained staff with 10 years of service conducted the questionnaire and physical examination in each selected area to ensure strict quality and minimize potential changes. Third, compared to the participants from hospital populations or undergoing physical examinations, in the current study, the participants were ordinary general populations residing in Rongchang, which was more representative. Despite these strengths, it is crucial to acknowledge the limitations of our study. First, as a cross-sectional study, it cannot establish causality between famine exposure and hyperuricemia. Second, consistent with other famine studies in China, the birthdate was used to define the exposure cohort, but we do not know the severity of the famine that participants went through. Third, in the current study, the lack of personal dietary information and family history of hyperuricemia might limit the ability to fully account for potential confounding factors related to hyperuricemia risk. Fourth, we did not consider the regional famine intensity during 1959–1962 on hyperuricemia at a county level like previous studies (50, 51), as there is no record or estimation of regional CSSI and EMR during a famine in Rongchang. Finally, younger populations born after the famine served as controls in our study, potentially posing challenges in distinguishing between age-related effects and those attributed to famine exposure (51). Nevertheless, we performed an age balance analysis, guided by previous research (51, 52), and we found that age did not exert a significant impact on our analysis.

## Conclusion

Taken together, women exposed to the Chinese famine during fetal, childhood, and adolescence were positively associated with hyperuricemia. The effect of famine on hyperuricemia might be intensified by the presence of dyslipidemia, abdominal obesity, and overweight/obesity in exposed women.

## Data availability statement

The raw data supporting the conclusions of this article will be made available by the authors, without undue reservation.

## Ethics statement

The studies involving humans were approved by the Ethics Committee of Rongchang Center for Disease Control and Prevention (no. RCJK20180023) and the Central Ethics Committee at the China National Center for Cardiovascular Disease (no. 2014–574). The studies were conducted in accordance with the local legislation and institutional requirements. The participants provided their written informed consent to participate in this study.

## Author contributions

HX: Conceptualization, Data curation, Formal analysis, Investigation, Methodology, Project administration, Resources, Software, Writing—original draft, Writing—review & editing. DL: Formal analysis, Investigation, Writing—original draft, Writing—review & editing. DT: Investigation, Project administration, Writing—review & editing. FM: Investigation, Methodology, Software, Writing—review & editing.
