# Supplementary material for: Exposure to Chinese famine and the risk of hyperuricemia in later life: a population-based cross-sectional study
Source: Front Nutr. 2024 Jan 17;11:1266817. doi: 10.3389/fnut.2024.1266817 (PMC10828035; doi:10.3389/fnut.2024.1266817)
Supplement: Supplementary file 1 [file Data_Sheet_1.PDF]

Supplementary Table 1:Univariate logistic analysis of different influencing factors associated with hyperuricemia

Variables	β	*S.E*	waldχ^2	*P*	*OR*	95%*CI*
Gender						
Males	-0.051	0.060	0.743	0.389	0.950	0.845~1.068
Females					Ref.	
Age in survey	0.018	0.003	30.585	＜0.001	1.018	1.012~1.025
Education level						
Primary school or below					Ref.	
Junior middle school	-0.022	0.075	0.086	0.770	0.978	0.844~1.134
High school or above	-0.168	0.075	5.085	0.024	0.845	0.730~0.978
Marital status						
Married/cohabitated	-0.068	0.084	0.661	0.416	0.934	0.792~1.101
Others					Ref.	
Job conditions						
Farmers	0.020	0.061	0.105	0.746	1.020	0.905~1.149
Others					Ref.	
Average yearly income，yuan						
＜20000					Ref.	
20001 to 59999	-0.294	0.072	16.641	＜0.001	0.745	0.647~0.858
≥60000	0.188	0.073	6.688	0.010	1.207	1.047~1.392
Smoking						
Never					Ref.	
Former/Current	-0.035	0.075	0.214	0.643	0.966	0.835~1.118
Drinking						
Never					Ref.	
Former/Current	-0.102	0.060	2.878	0.090	0.903	0.803~1.016
Physical activity						
Light					Ref.	
Moderate	-0.028	0.087	0.104	0.747	0.972	0.820~1.153
Vigorous	-0.291	0.075	15.137	＜0.001	0.747	0.645~0.865
Hypertension						
No					Ref.	
Yes	0.530	0.062	73.387	＜0.001	1.699	1.505~1.918
Diabetes						

No					Ref.	
Yes	-0.178	0.087	4.191	0.041	0.837	0.706~0.992
Dyslipidemia						
No					Ref.	
Yes	0.621	0.063	96.597	＜0.001	1.860	1.644~2.106
BMI group , Kg/m2						
＜24					Ref.	
≥24	0.451	0.065	48.855	＜0.001	1.570	1.384~1.782
Abdominal obesity						
No					Ref.	
Yes	0.595	0.061	95.676	＜0.001	1.813	1.609~2.042
famine exposure group						
Non-exposed					Ref.	
Fetal- Exposed	0.447	0.085	2.366	＜0.001	1.564	1.327~1.847
Childhood- Exposed	0.453	0.086	2.428	＜0.001	1.573	1.329~1.862
Adolescent- Exposed	0.469	0.082	32.626	＜0.001	1.598	1.361~1.877

Supplementary Table 2: Multivariate logistic analysis of different influencing factors associated with hyperuricemia

Variables	β	*S.E*	waldχ^2	*P*	*OR*	95%*CI*
Age in survey	-0.001	0.013	0.002	0.966	0.999	0.975~1.024
Average yearly income，yuan						
＜20000					Ref.	
20001 to 59999	-0.231	0.075	9.543	0.002	0.794	0.686~0.919
≥60000	0.228	0.076	8.904	0.003	1.256	1.081~1.459
Physical activity						
Light					Ref.	
Moderate	-0.058	0.090	0.416	0.519	0.944	0.791~1.125
Vigorous	-0.187	0.080	5.431	0.020	0.830	0.709~0.971
Hypertension						
No					Ref.	
Yes	0.391	0.066	34.974	＜0.001	1.479	1.299~1.684
Diabetes						
No					Ref.	
Yes	-0.501	0.091	30.036	＜0.001	0.606	0.506~0.725
Dyslipidemia						
No					Ref.	
Yes	0.576	0.066	75.631	＜0.001	1.779	1.562~2.025
BMI group , Kg/m2						
＜24					Ref.	
≥24	0.248	0.070	12.429	＜0.001	1.282	1.117~1.471
Abdominal obesity						
No					Ref.	
Yes	0.452	0.067	45.569	＜0.001	1.571	1.378~1.792
famine exposure group						
Non-exposed					Ref.	
Fetal- Exposed	0.193	0.089	0.002	＜0.001	1.213	1.019~1.444
Childhood- Exposed	0.225	0.094	1.383	＜0.001	1.252	1.042~1.506
Adolescent- Exposed	0.245	0.093	6.991	＜0.001	1.278	1.065~1.533

Supplementary Figure1: Simple linear regression analysis of the association between birth year and hyperuricemia

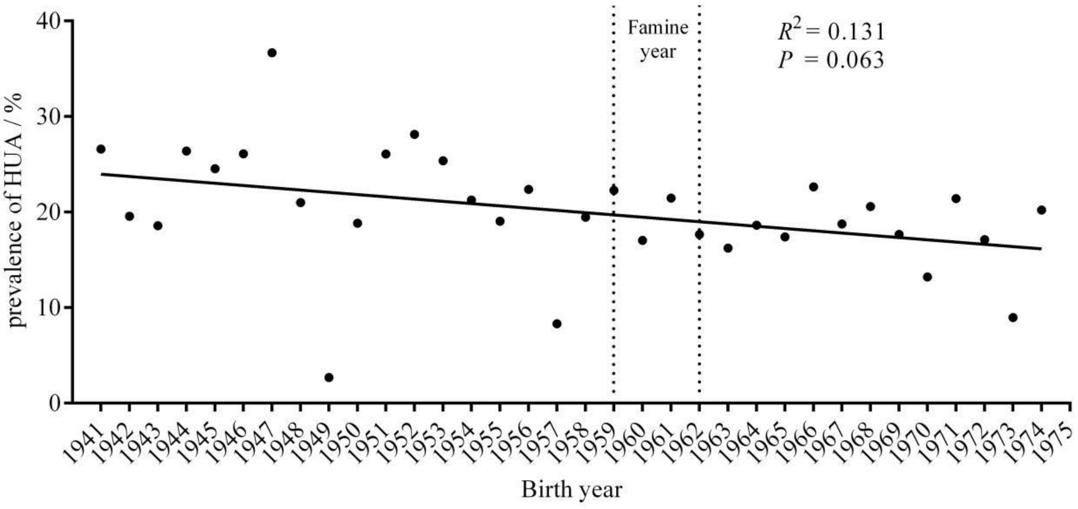

Supplementary Figure2: Odds ratios were calculated by comparing different exposed group to different age balance controls

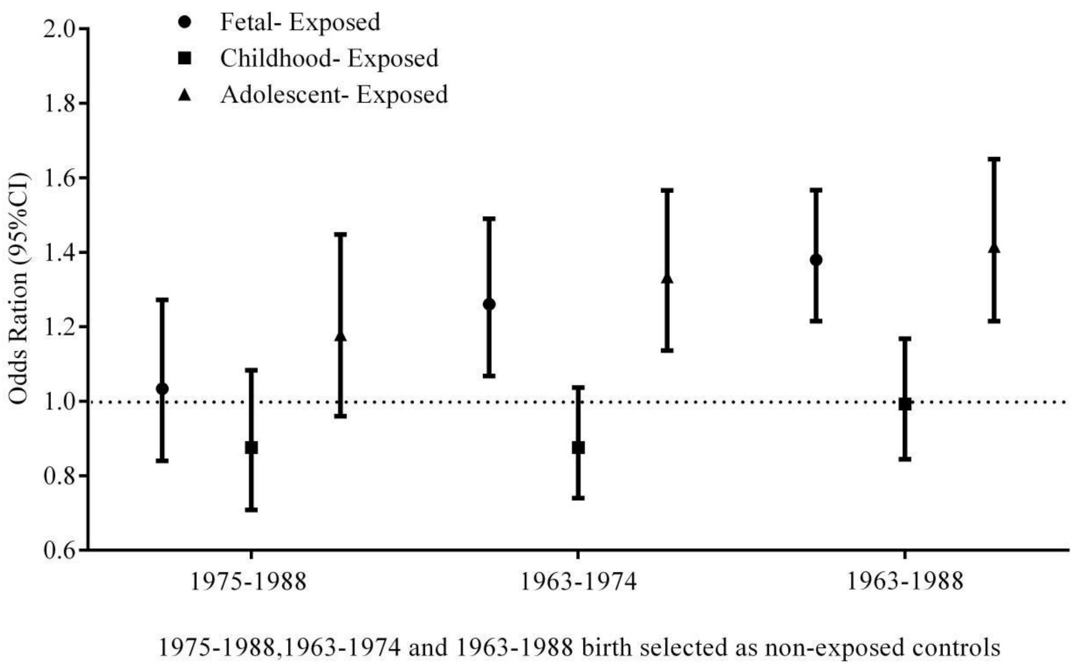

Supplementary Table 3: The association between fetal and childhood exposure to the Chinese famine and the risk of hyperuricemia compared with age-matched control groups

Variables	β	*S.E*	waldχ^2	*P*	*OR*	95%*CI*
Males						
Non-exposed					Ref.	
Fetal- Exposed	-0.527	0.113	21.611	0.000	0.590	0.473~0.737
Childhood- Exposed	-0.322	0.111	8.353	0.004	0.725	0.582~0.902
Females						
Non-exposed					Ref.	
Fetal- Exposed	0.265	0.099	7.121	0.008	1.304	1.073~1.585
Childhood- Exposed	0.255	0.102	1.282	0.258	1.290	1.057~1.576
